# Supplementary material for: SHARPIN Enhances Ferroptosis in Synovial Sarcoma Cells via NF-κB- and PRMT5-Mediated PGC1α Reduction
Source: Cancers (Basel). 2023 Jul 4;15(13):3484. doi: 10.3390/cancers15133484 (PMC10341212; doi:10.3390/cancers15133484)

Figure 1D

TFRC

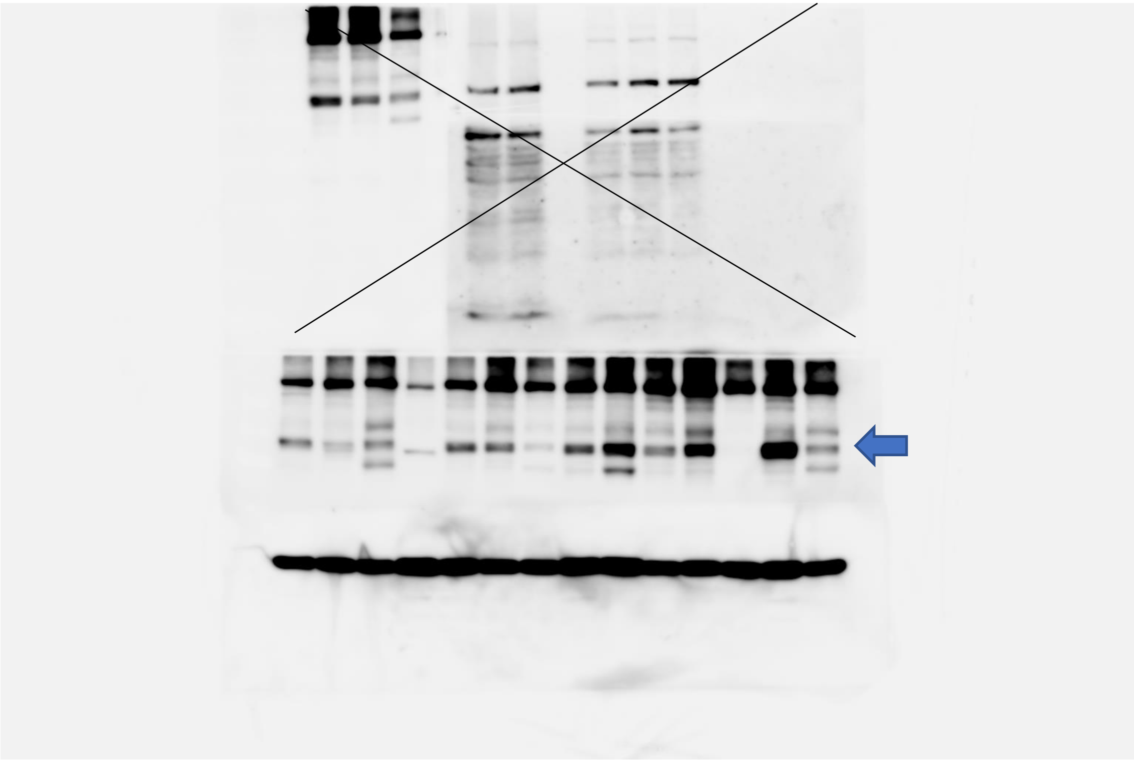

Figure 1D

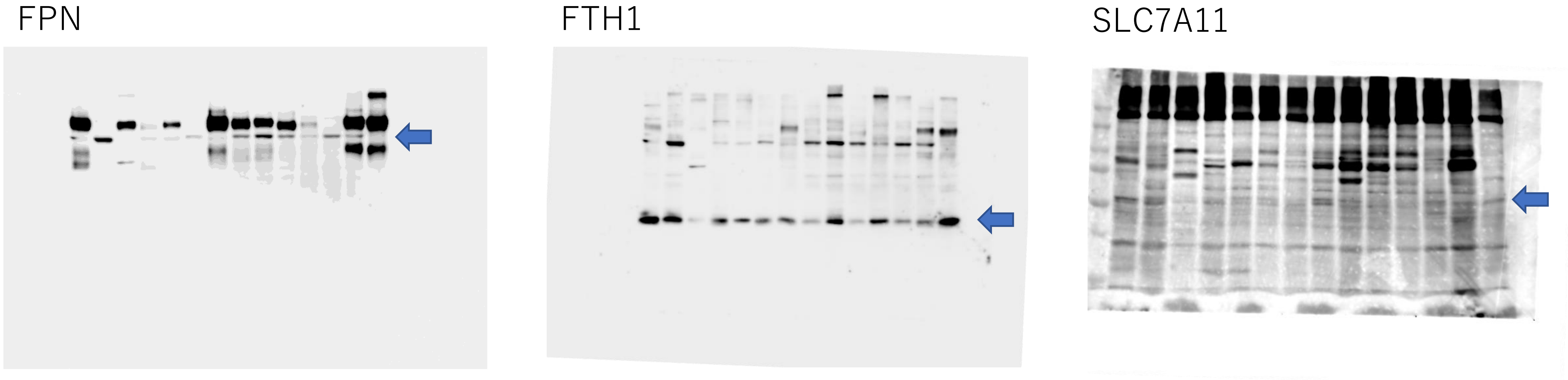

Figure 1D

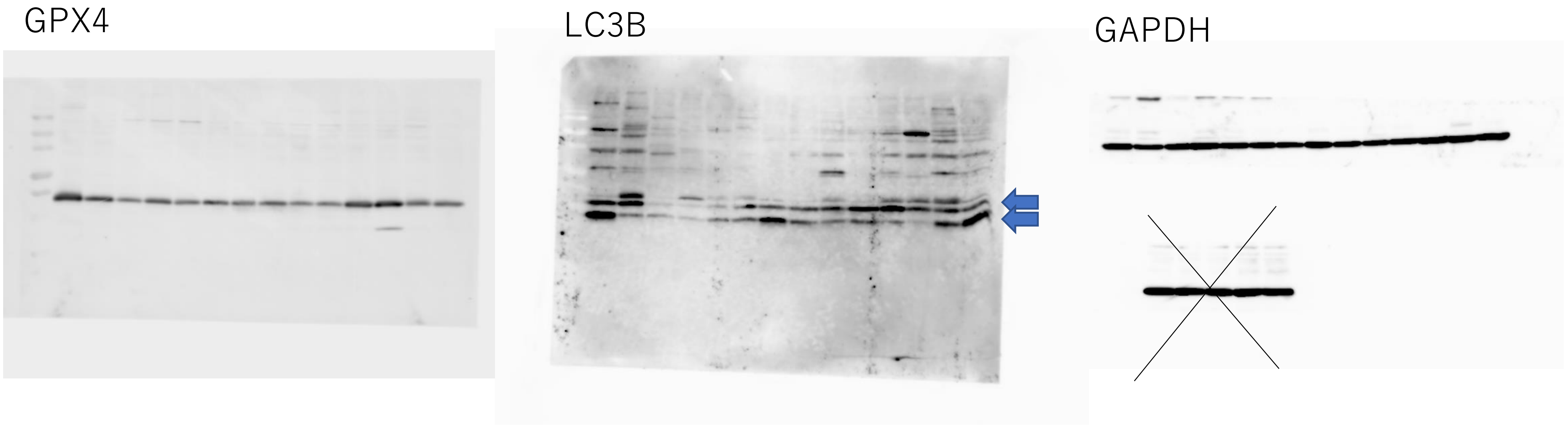

Figure 4E

PGC1a

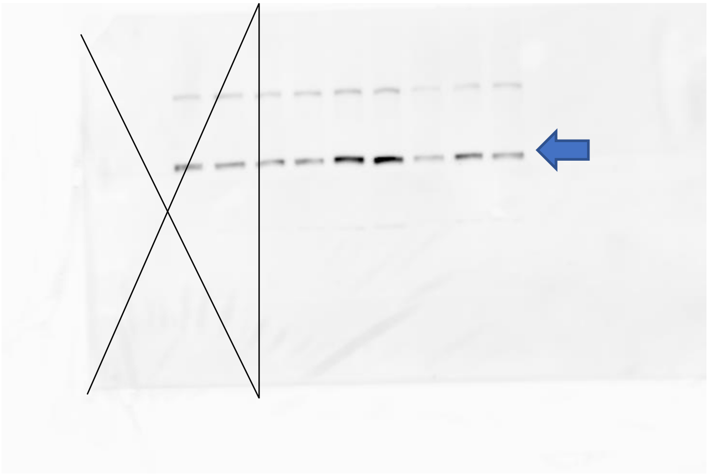

SLC7A11

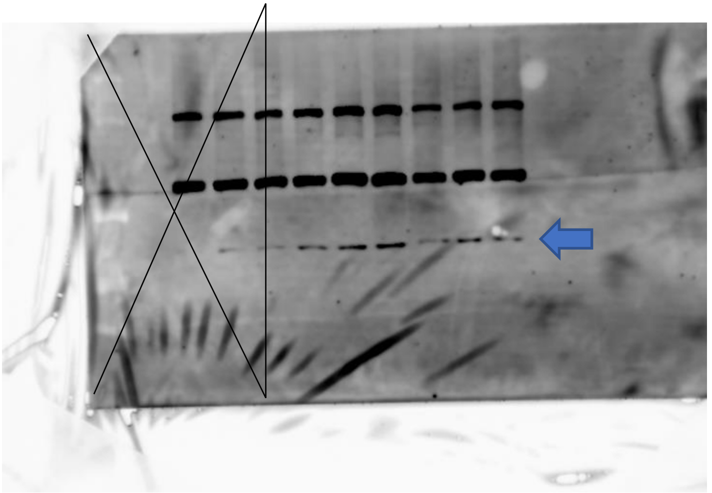

SHARPIN

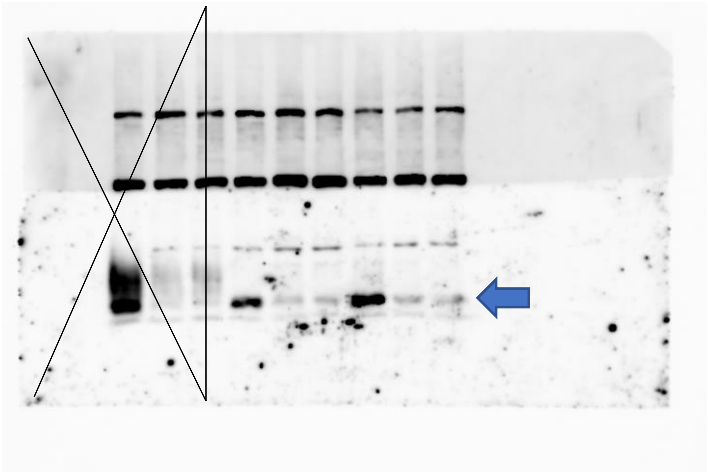

B-actin

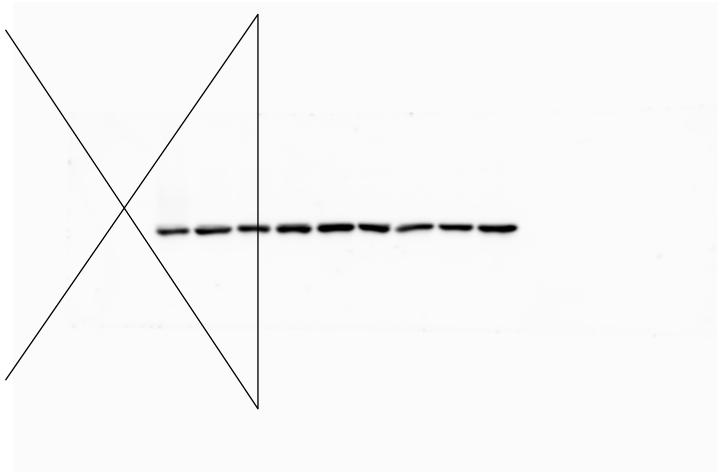

ComplexIII/V

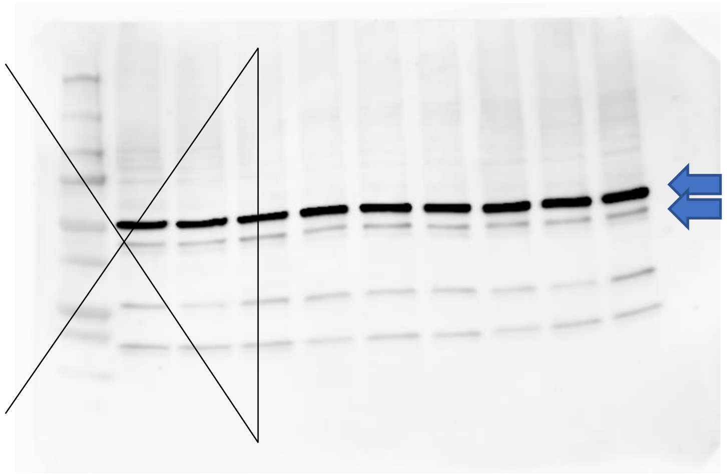

ComplexI

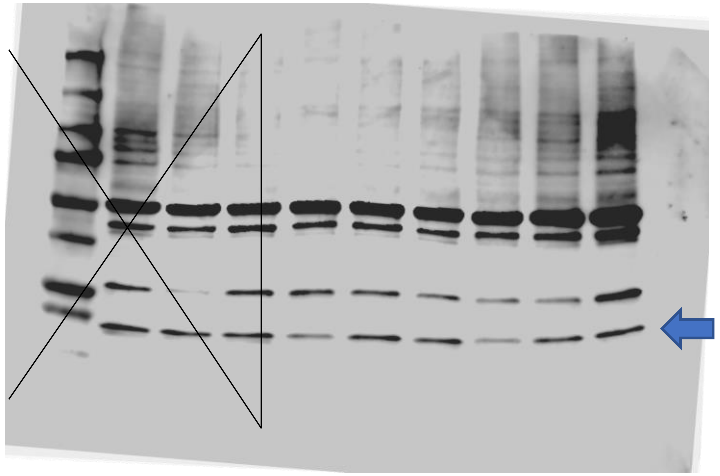

VDAC1/3

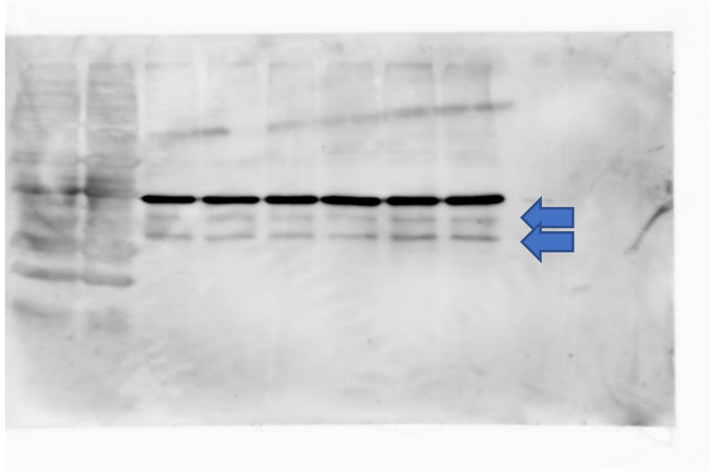

GAPDH

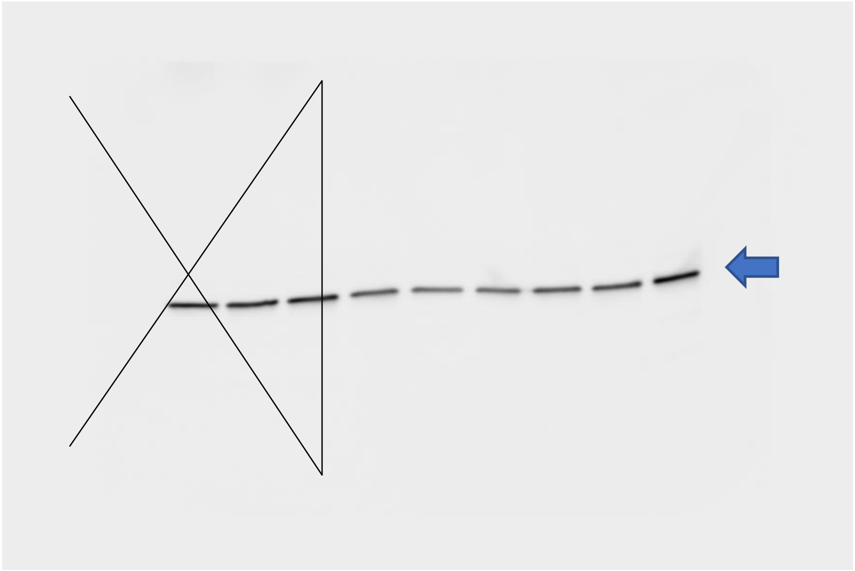

Parkin

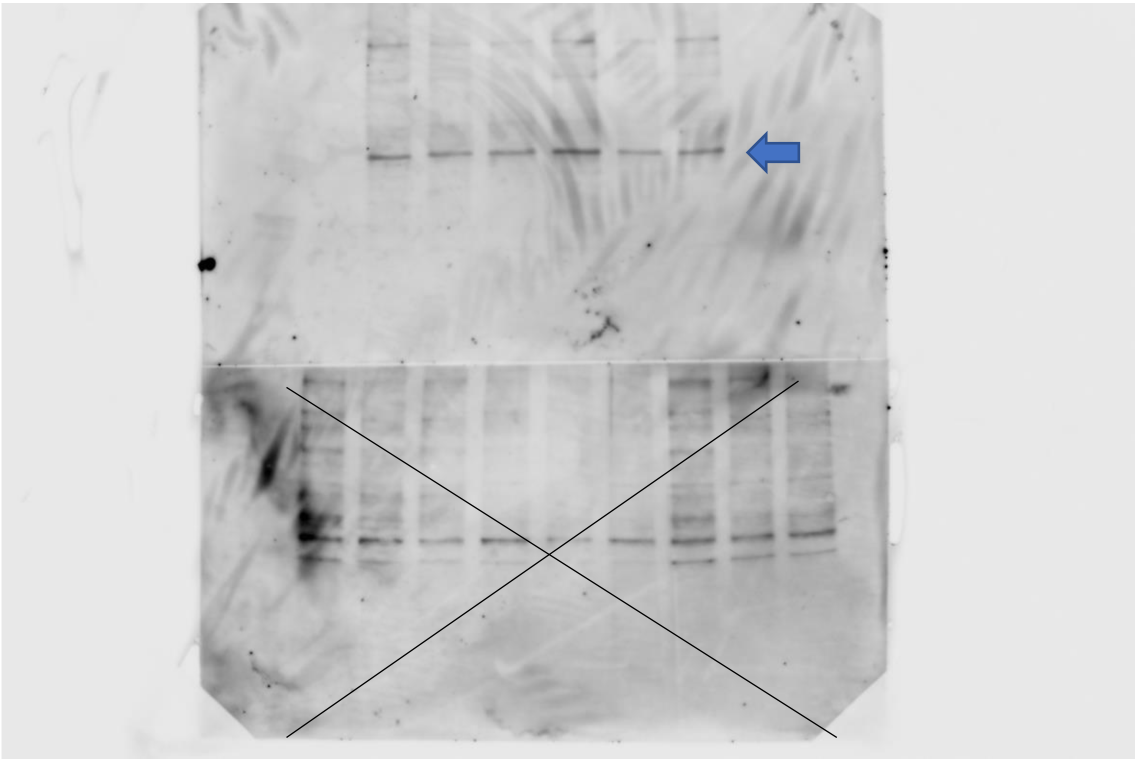

BNIP3L/NIX

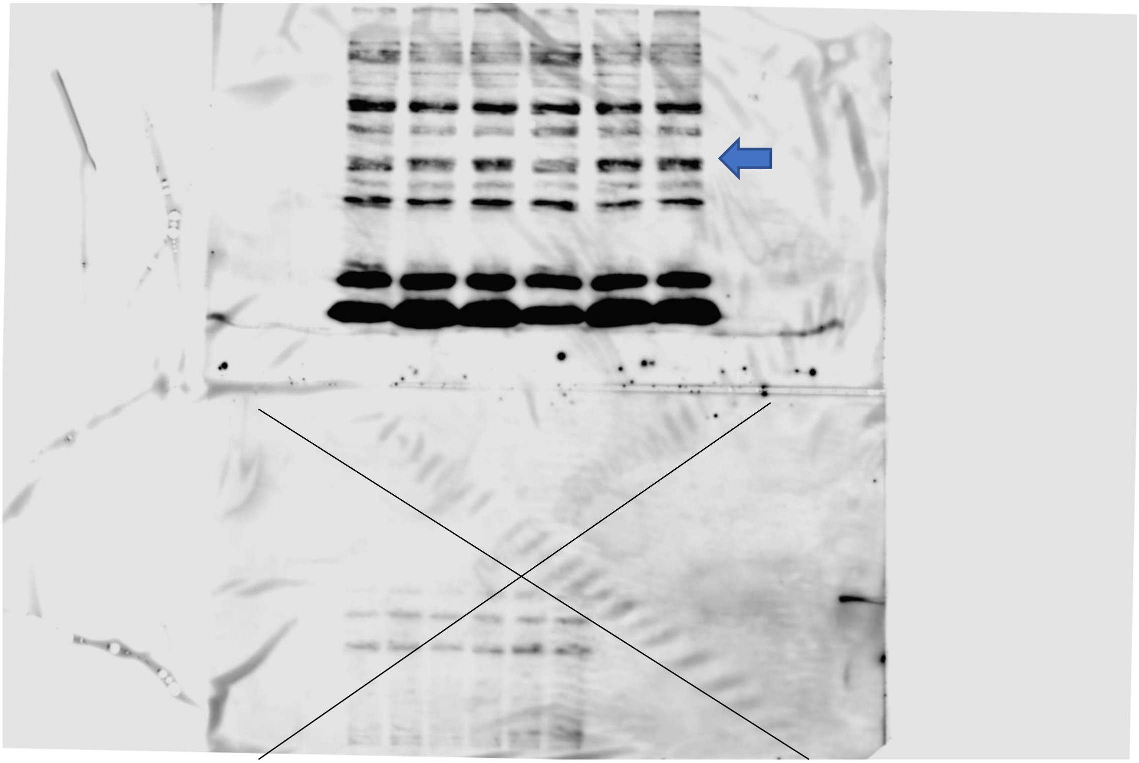

LC3B

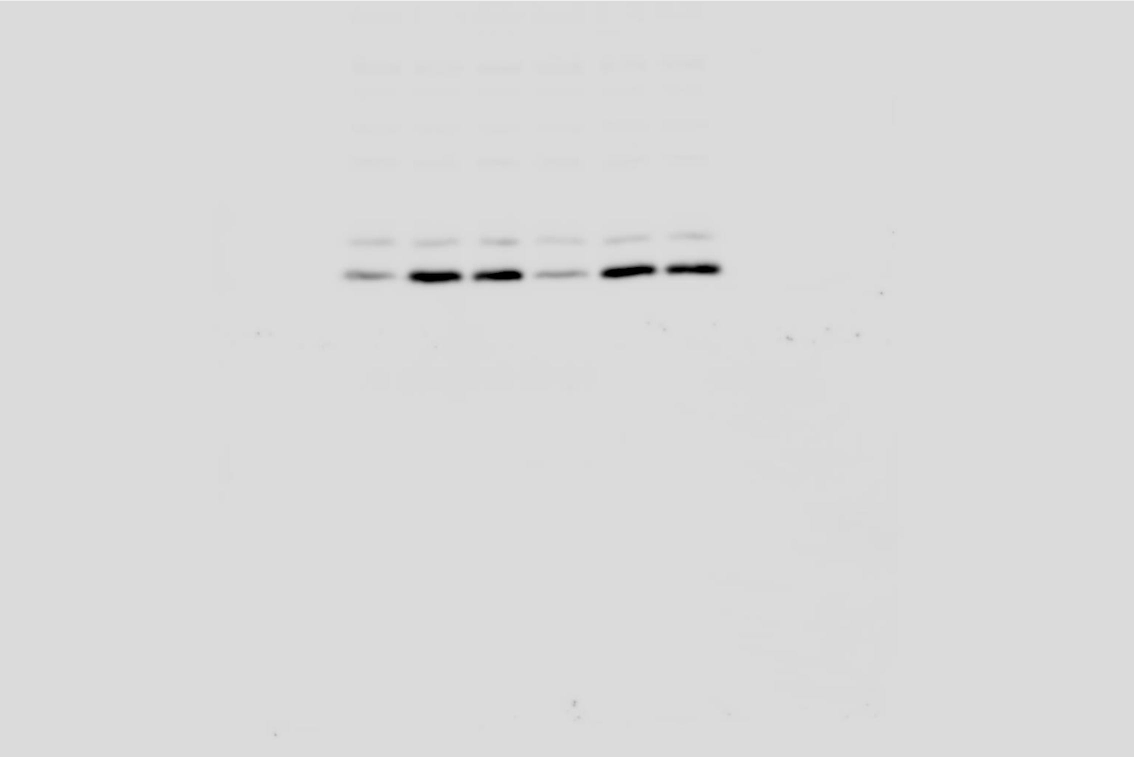

Figure 4G

Nrf2

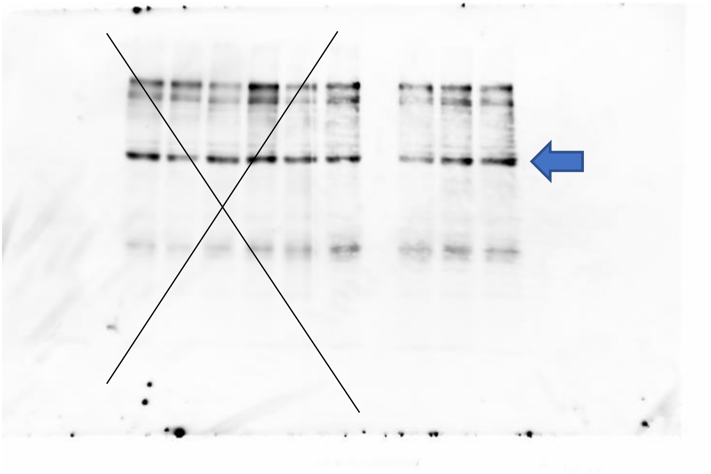

SHARPIN

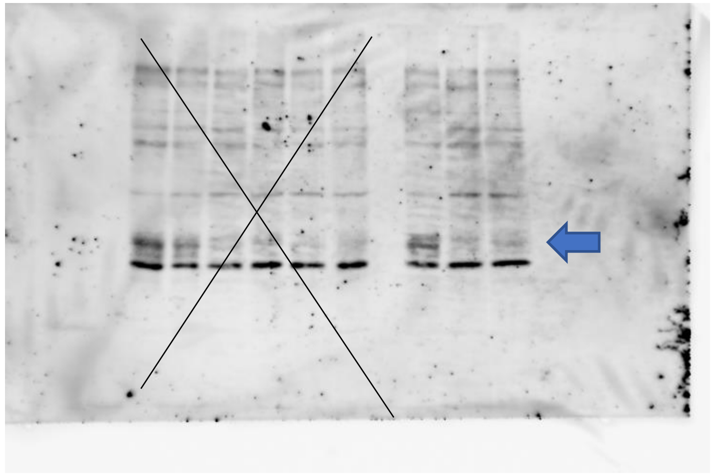

GAPDH

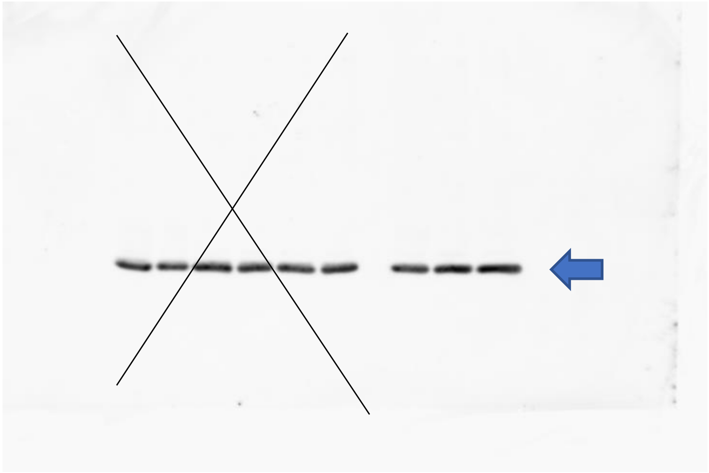

Figure 5B

SHARPIN

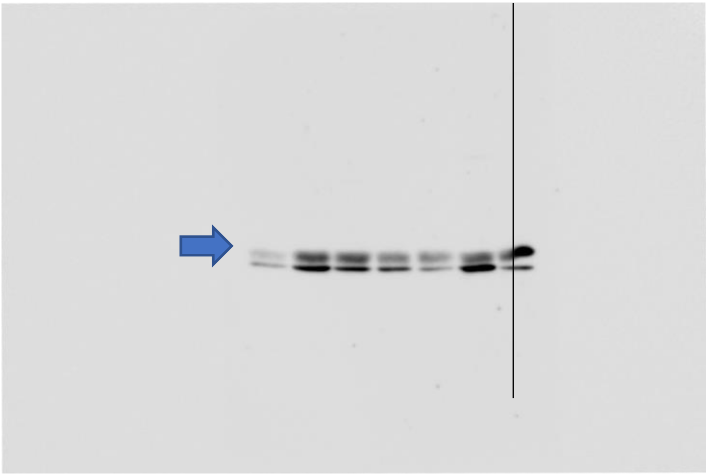

PRMT5

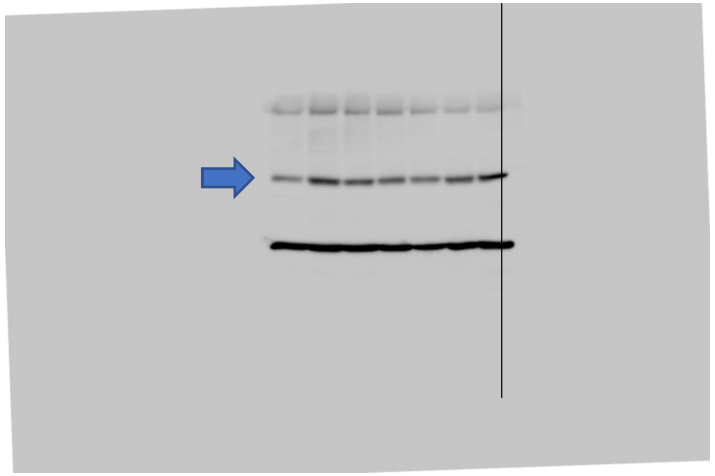

SDMA

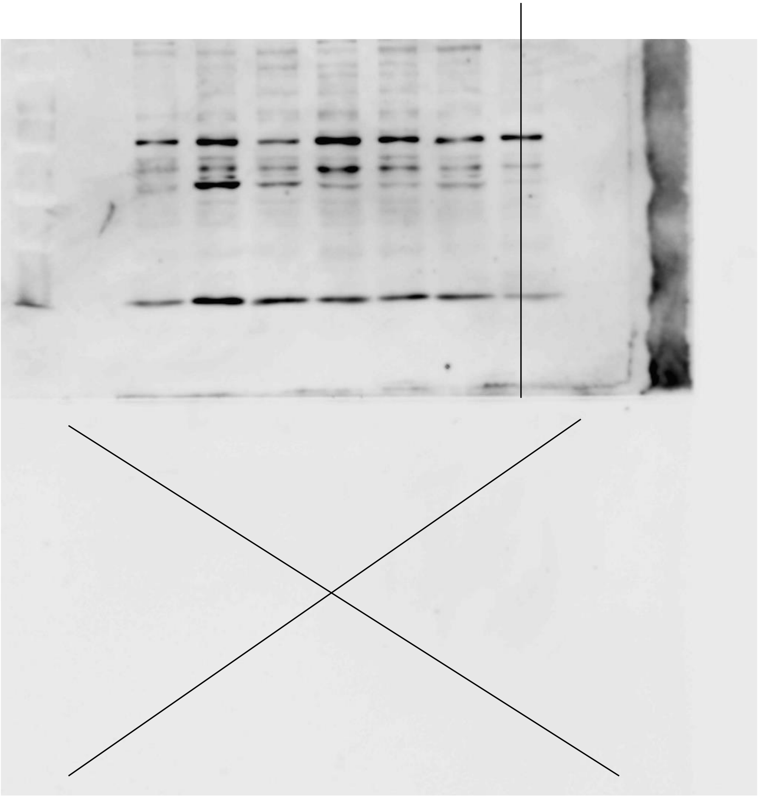

SOX10

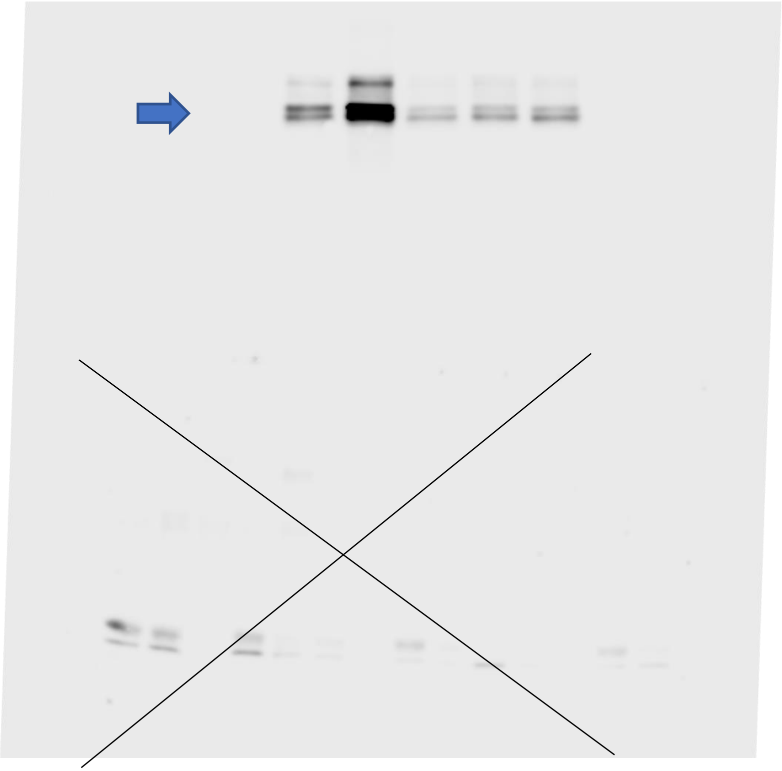

MITF

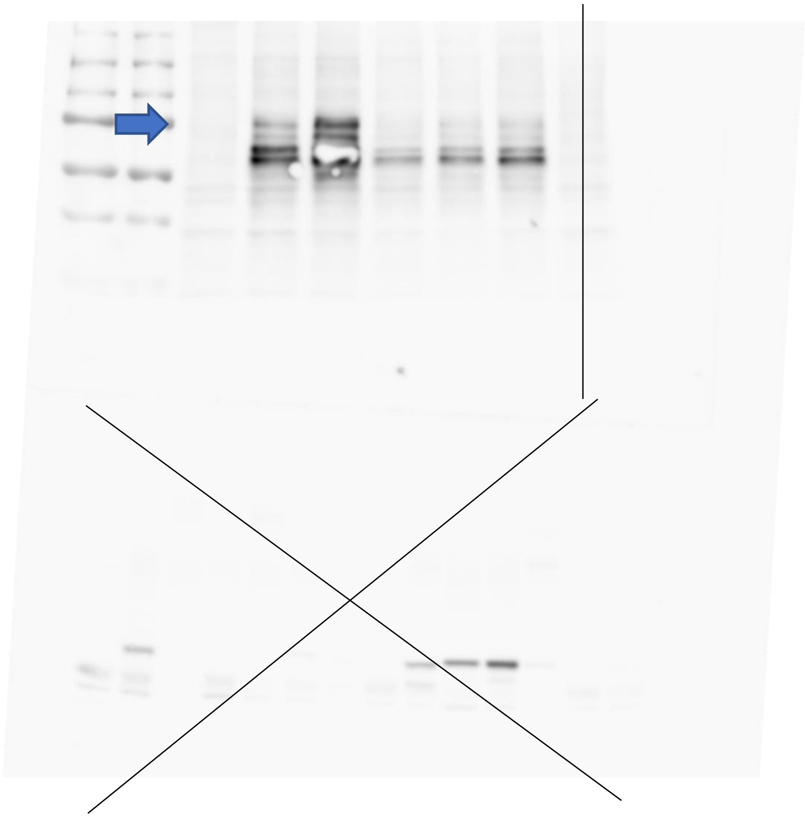

PGC1a

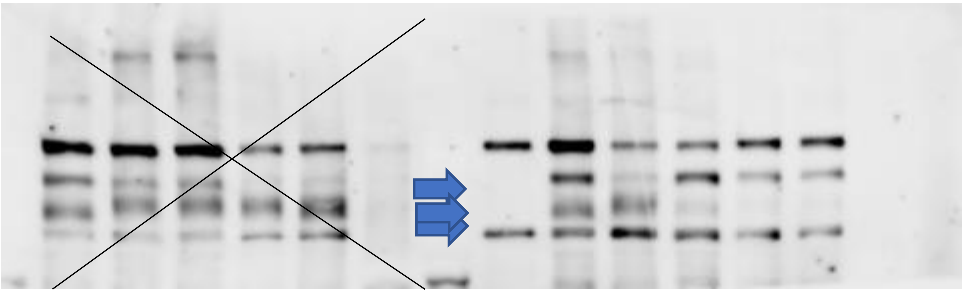

SLC7A11

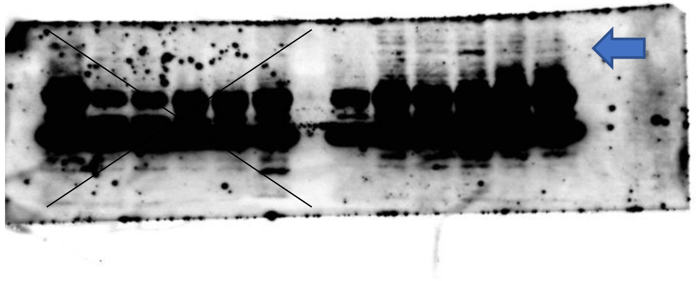

GAPDH

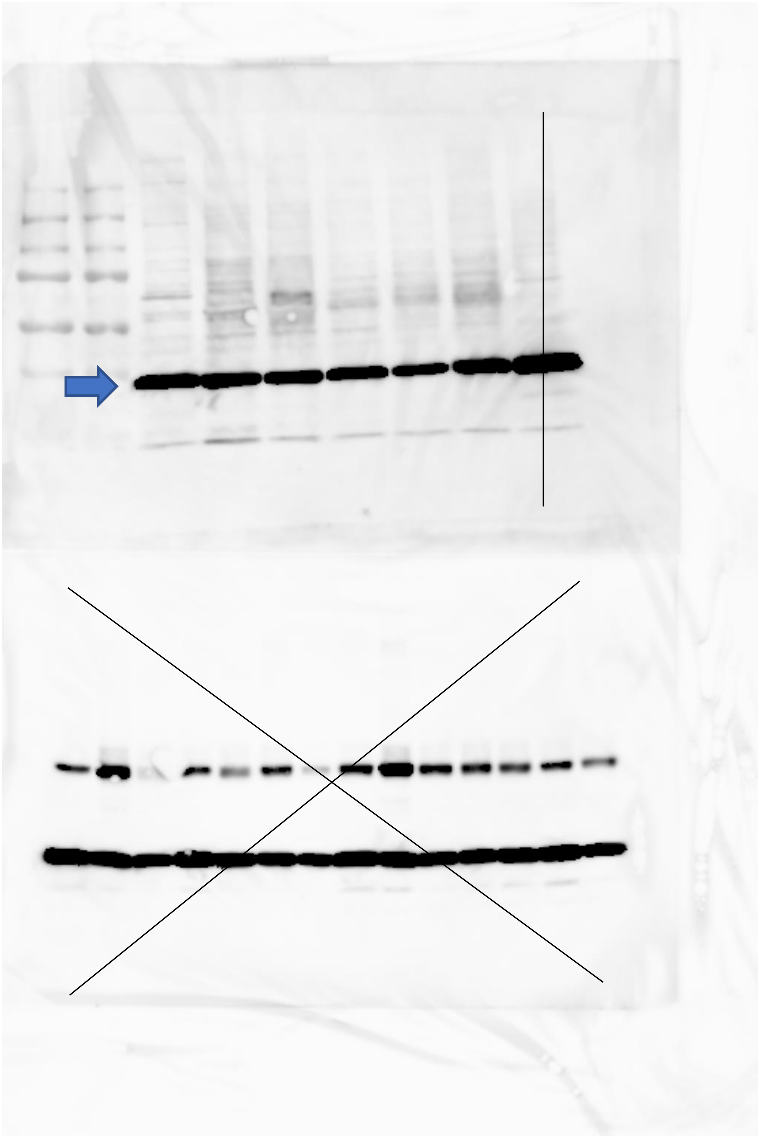

Figure 5D

SHARPIN

PGC1a

GAPDH

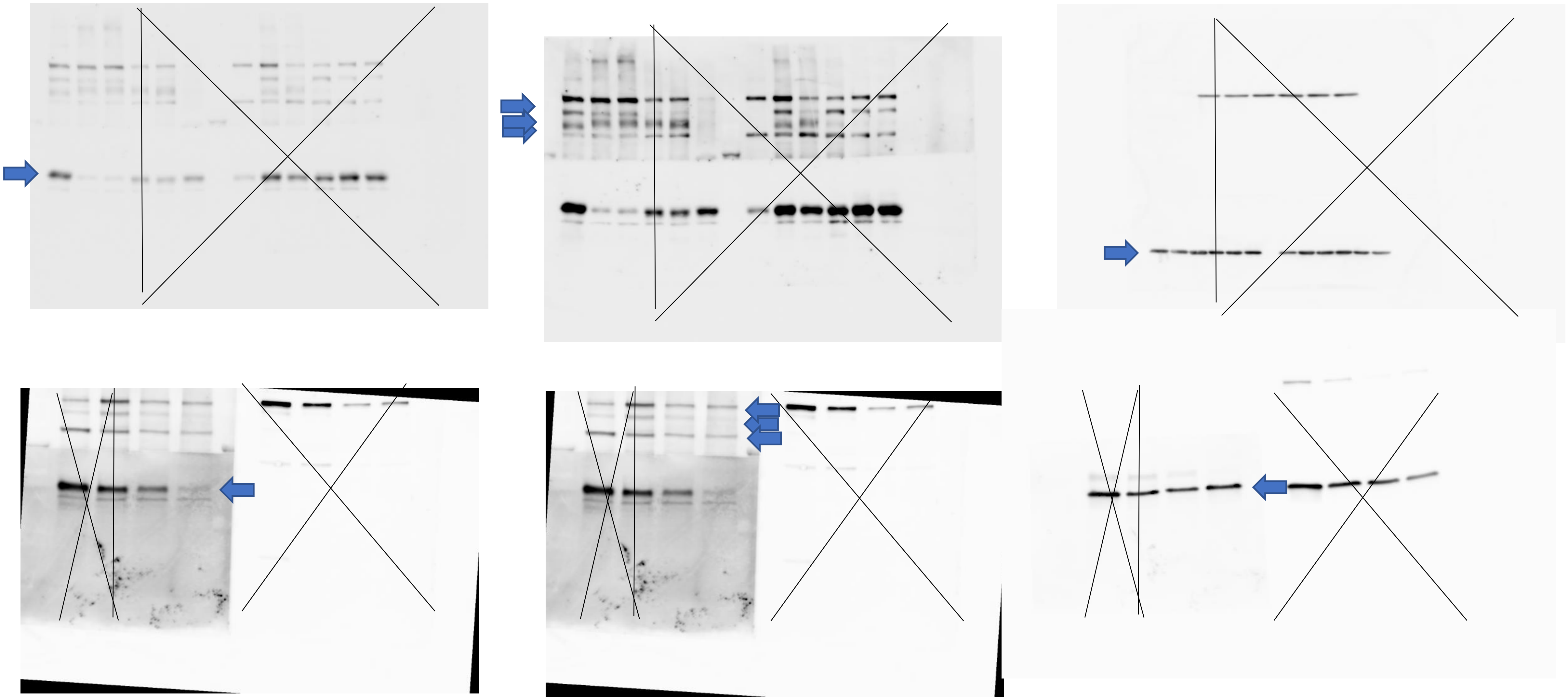

Figure 6A

SDMA

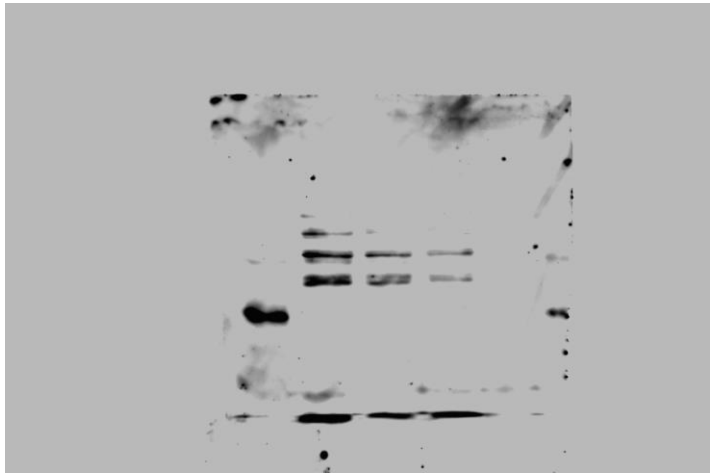

SHARPIN

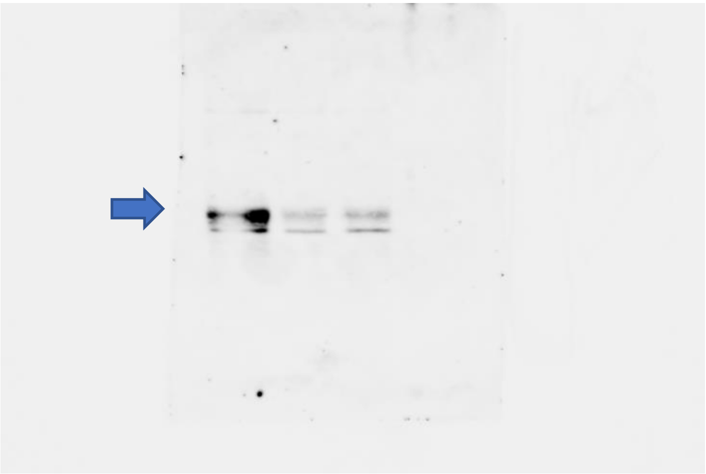

GAPDH

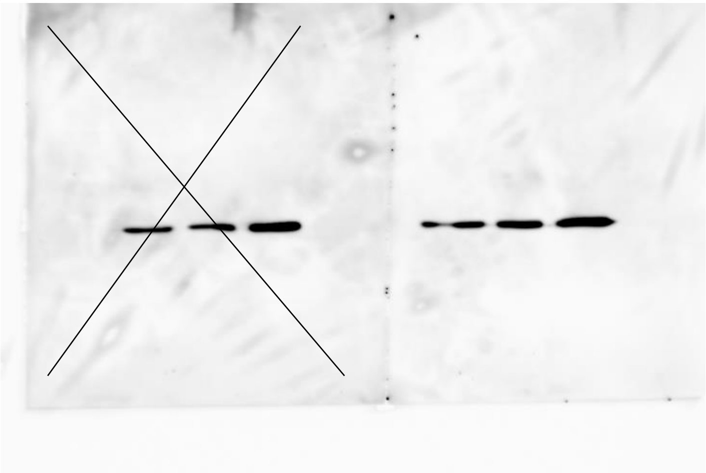

Figure 6B

PGC1a

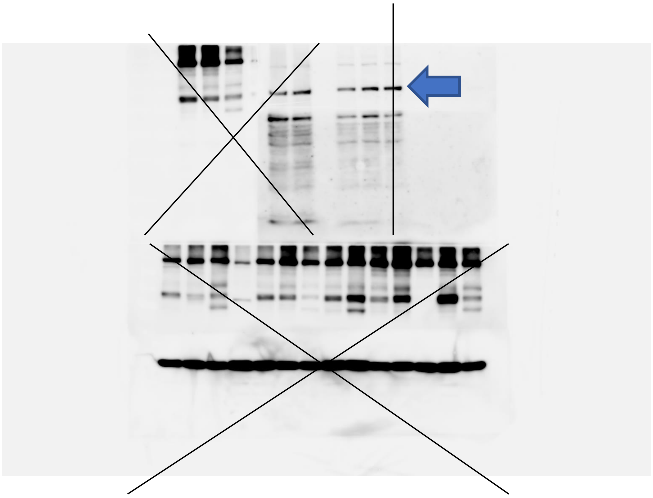

SDMA

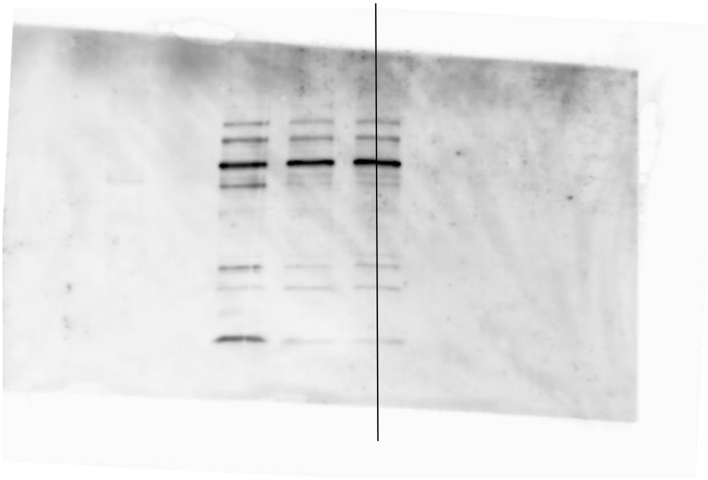

SLC7A11

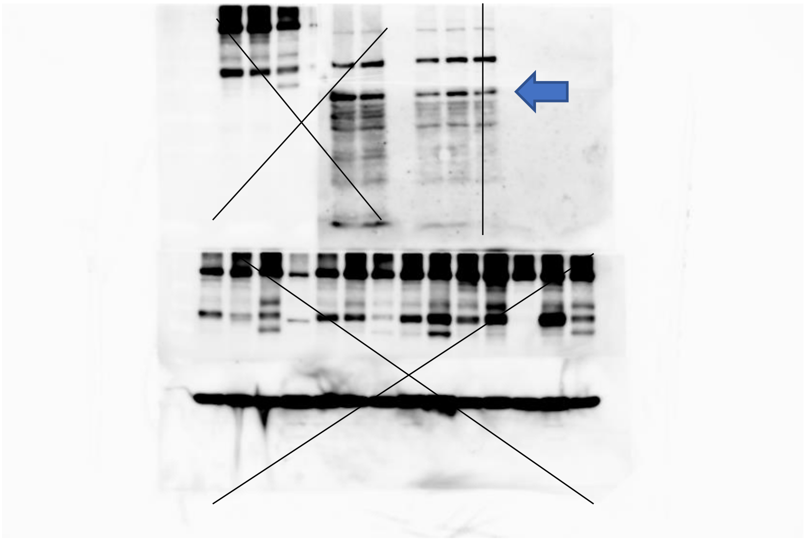

SHARPIN

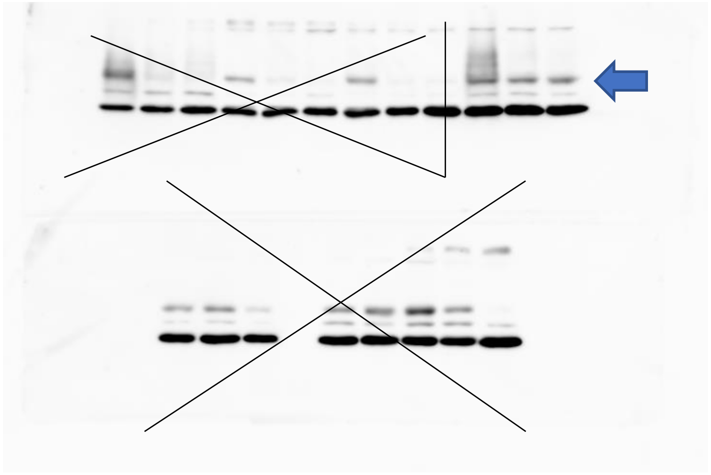

GAPDH

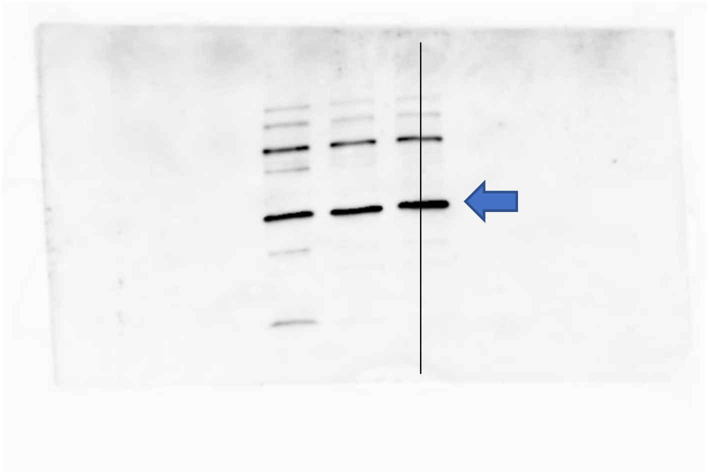

Figure 6C

PGC1a

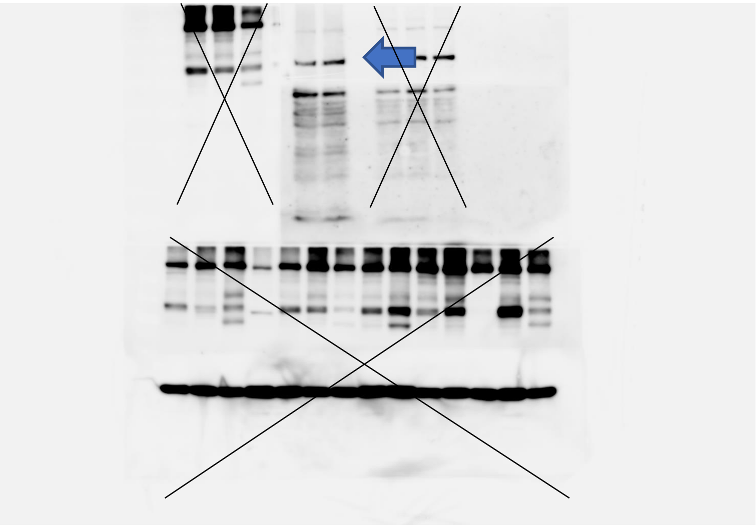

SLC7A11

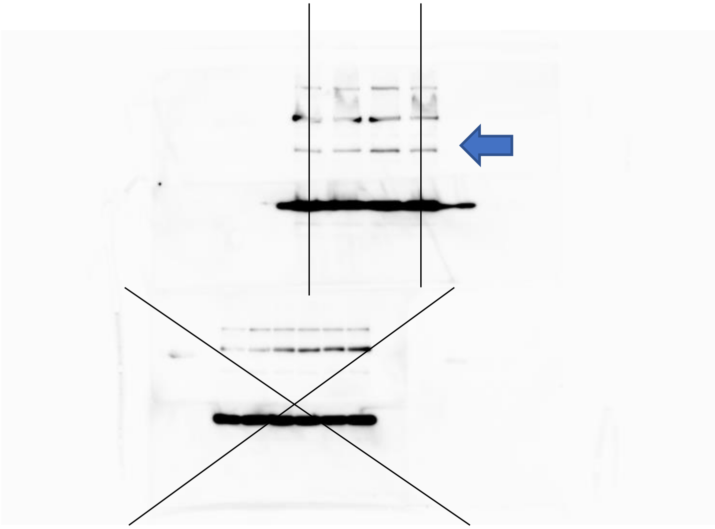

GAPDH

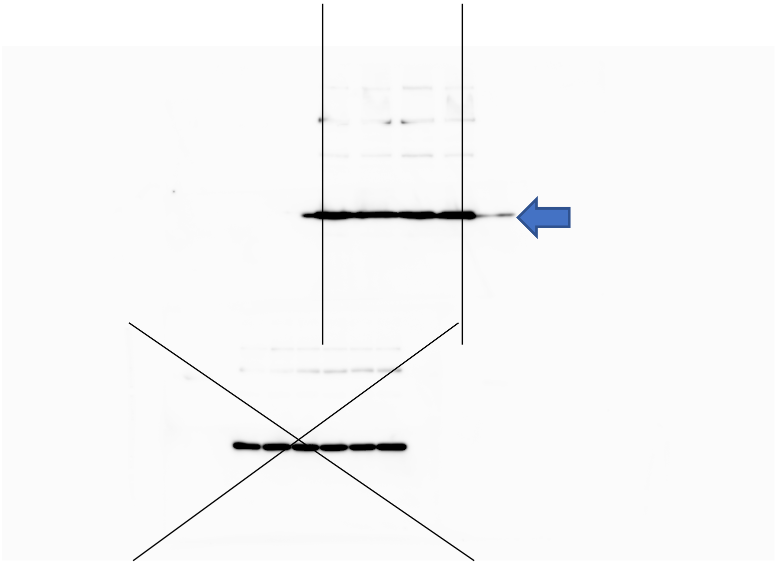

Figure S10

TFRC

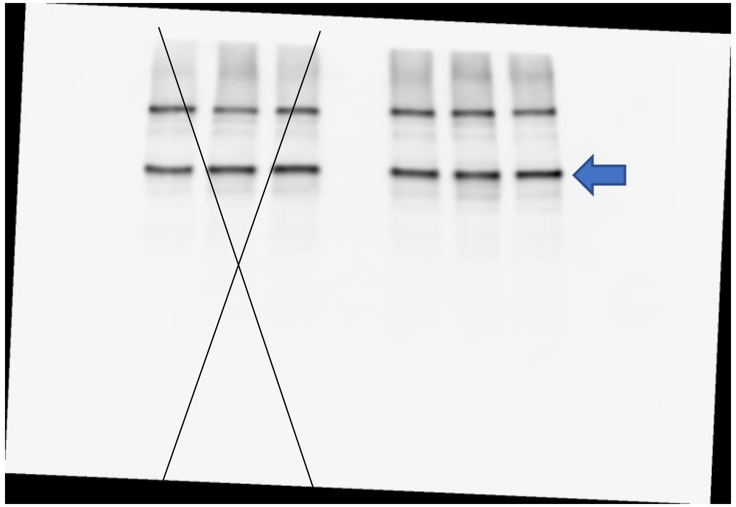

SHARPIN

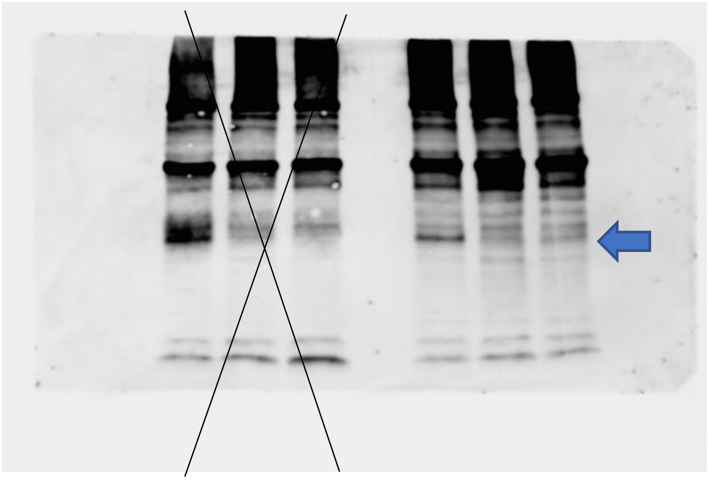

B-actin

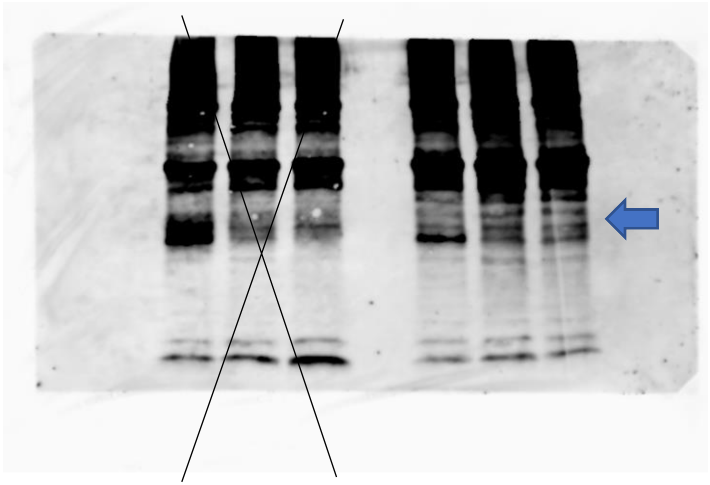

Figure S10

GPX4

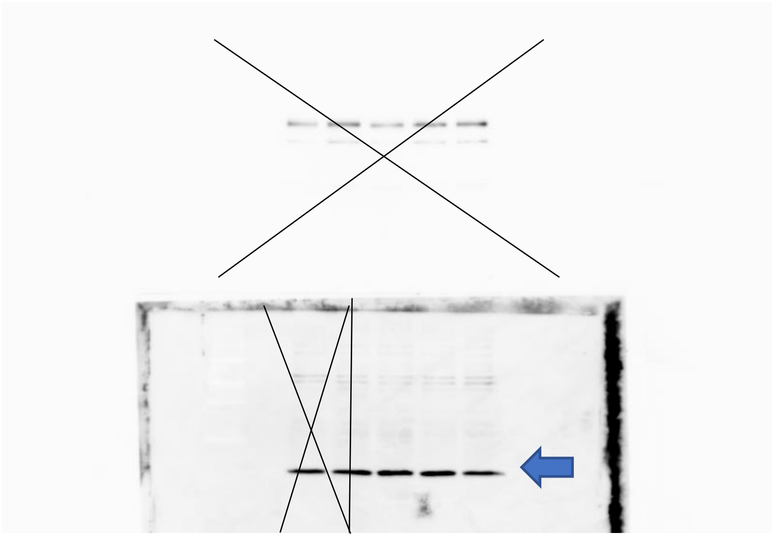

SHARPIN

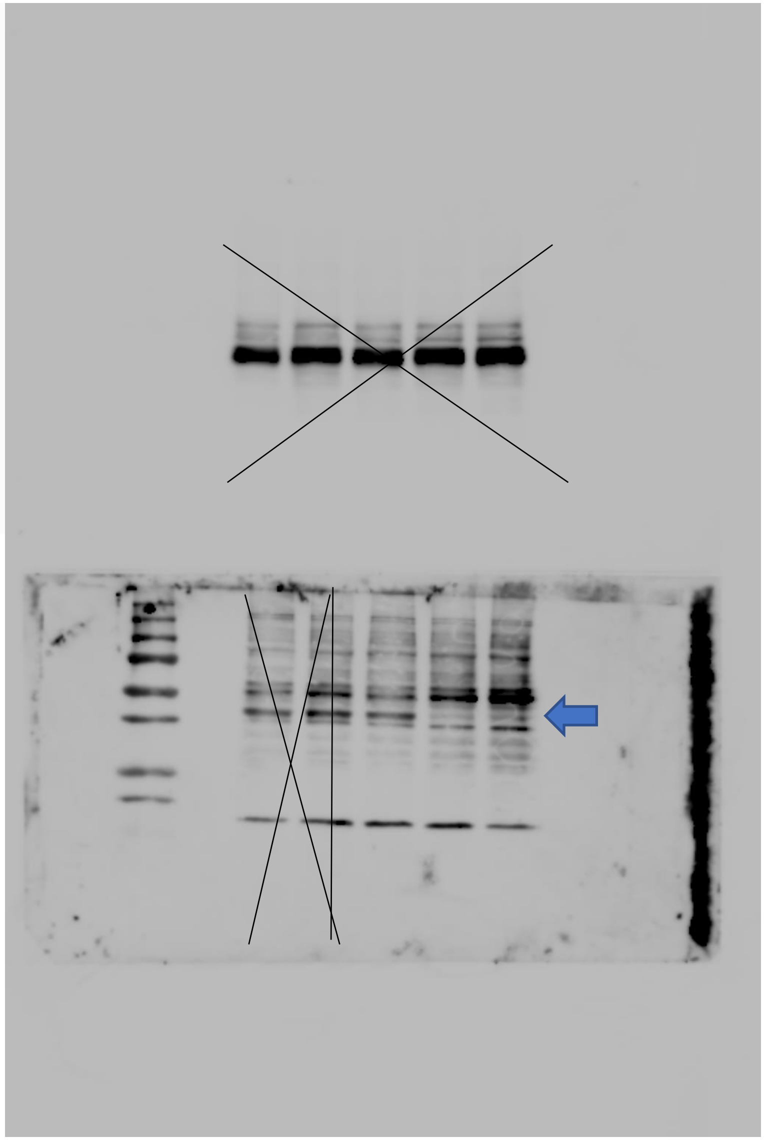

GAPDH

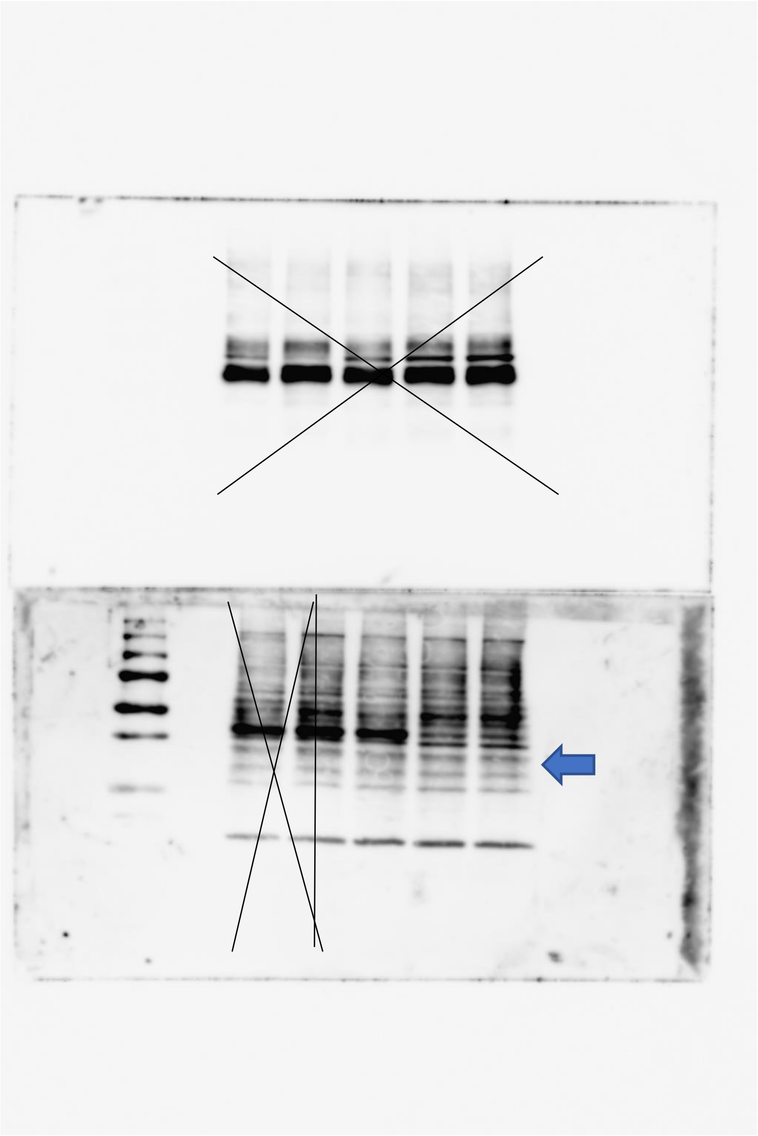

Figure S11

PGC1a

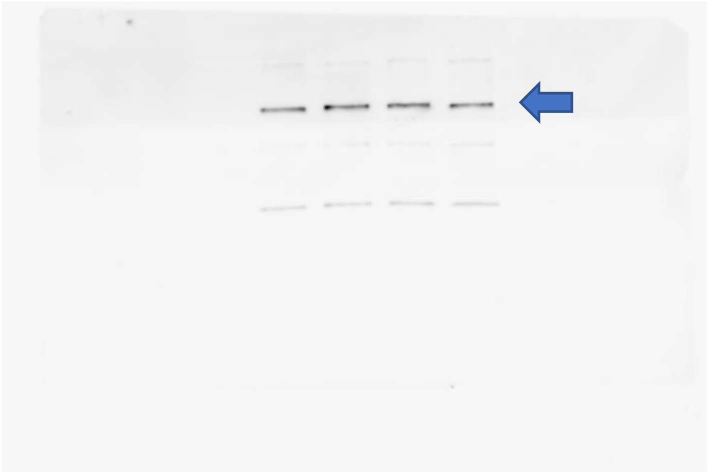

SLC7A11
GAPDH

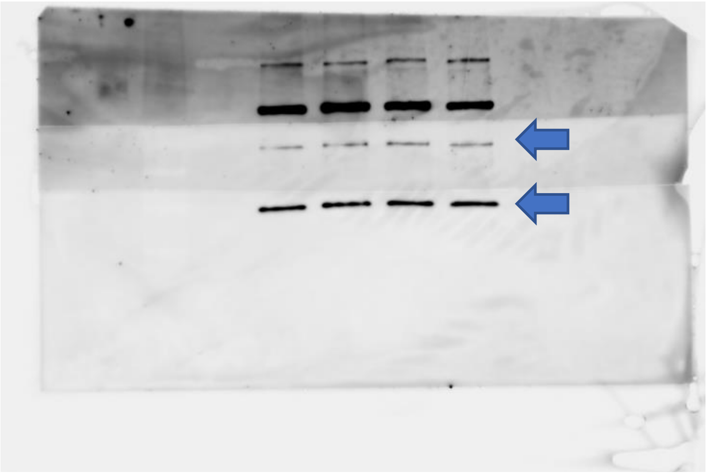

Figure S11

SLC7A11

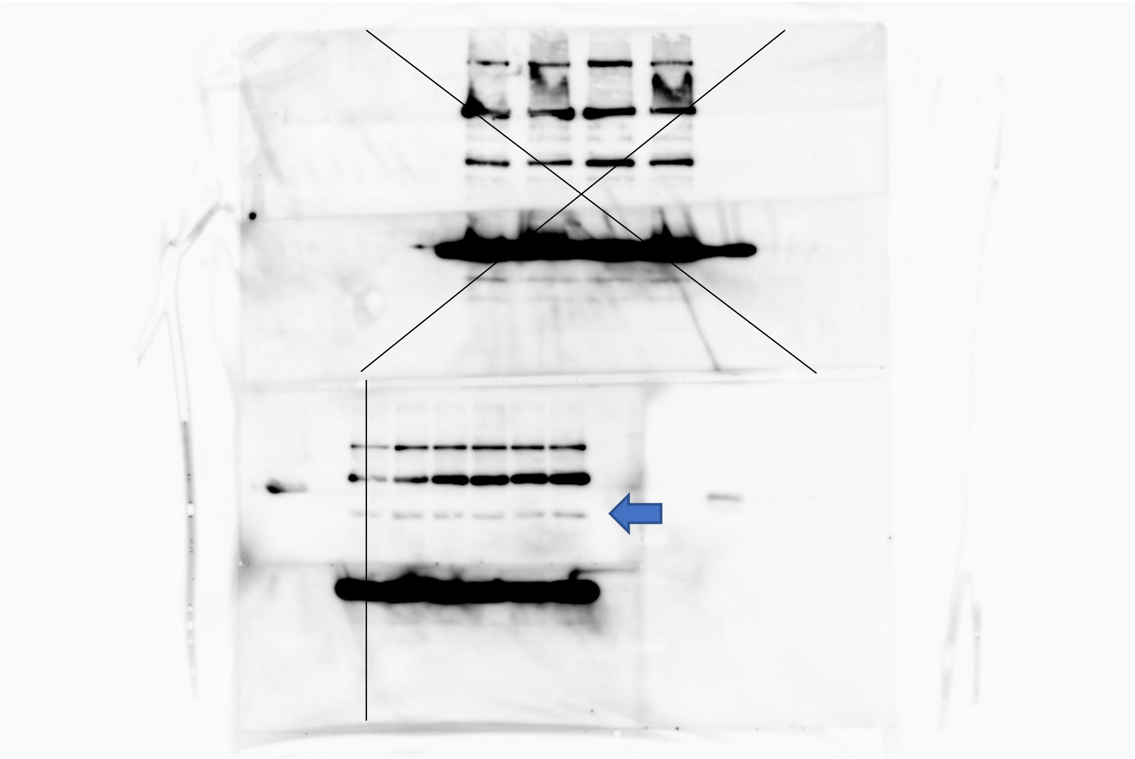

GAPDH

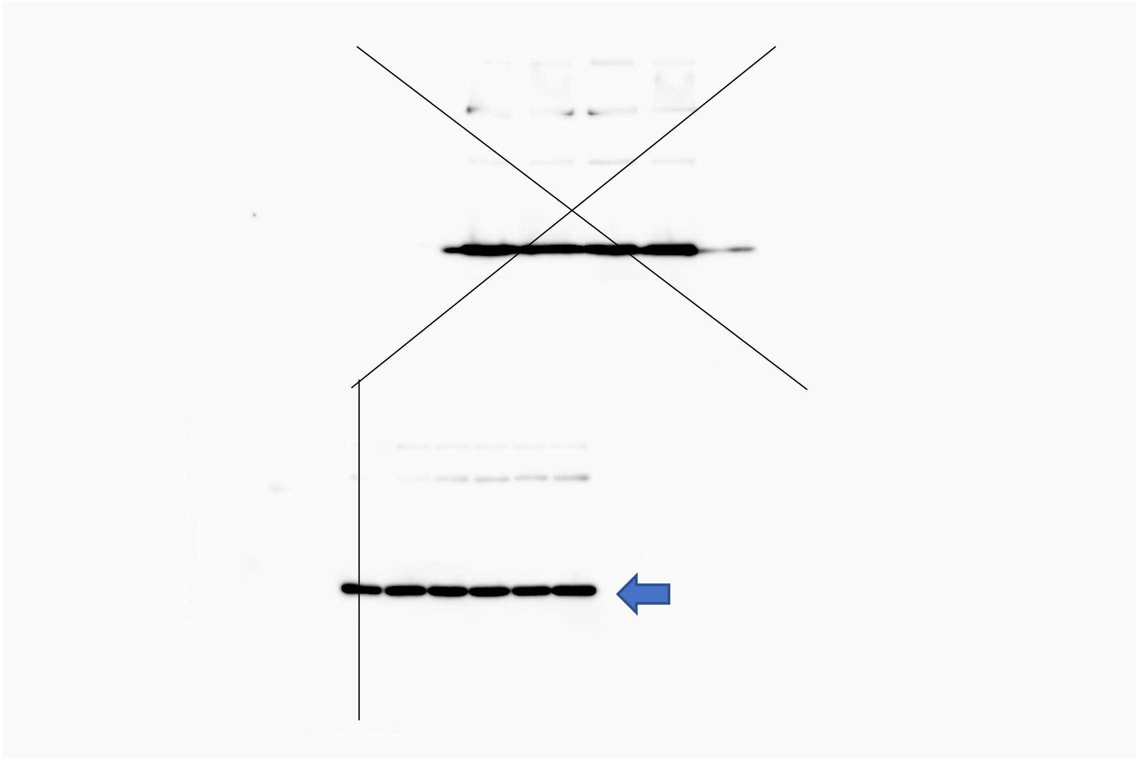

Supplement: Supplementary file 1 [file cancers-15-03484-s001.zip › File S1. original-images.pdf]
