# Supplementary material for: SHARPIN Enhances Ferroptosis in Synovial Sarcoma Cells via NF-κB- and PRMT5-Mediated PGC1α Reduction
Source: Cancers (Basel). 2023 Jul 4;15(13):3484. doi: 10.3390/cancers15133484 (PMC10341212; doi:10.3390/cancers15133484)
Supplement: Supplementary file 1 [file cancers-15-03484-s001.zip › Supple Figure1.pdf]

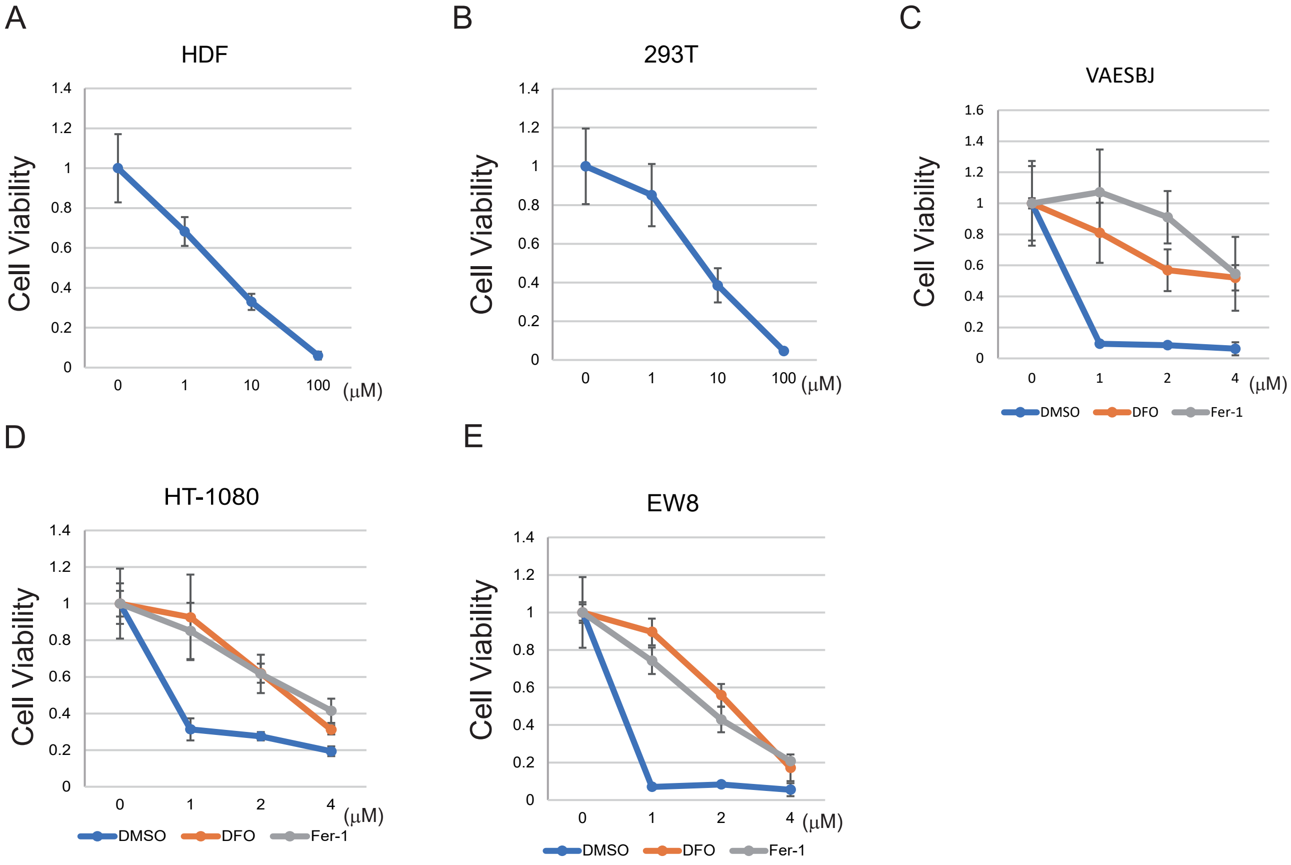

Figure S1. Viability assays of sarcoma and non-sarcoma cell lines following treatment with RSL3.
HDF (A) and the 293T (B), VAESBJ (C), HT-1080 (D), and EW8 (E) cell lines were treated with the indicated concentration of RSL3 for 24 hours. (C–E) DFO or Fer-1 was used to inhibit ferroptosis.
