# Supplementary material for: SHARPIN Enhances Ferroptosis in Synovial Sarcoma Cells via NF-κB- and PRMT5-Mediated PGC1α Reduction
Source: Cancers (Basel). 2023 Jul 4;15(13):3484. doi: 10.3390/cancers15133484 (PMC10341212; doi:10.3390/cancers15133484)
Supplement: Supplementary file 1 [file cancers-15-03484-s001.zip › Supple Figure10.pdf]

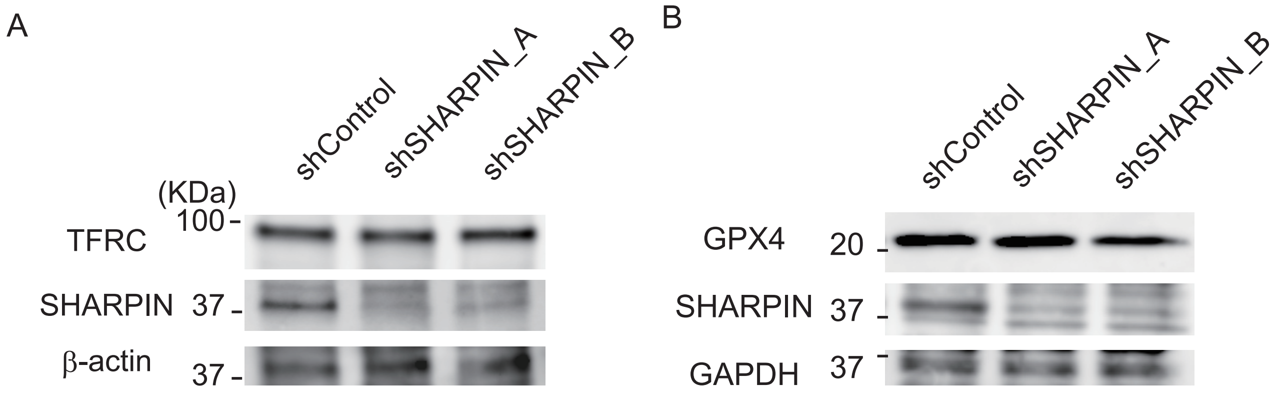

Figure S10. Knockdown of SHARPIN does not affect the expression of TFRC or GPX4 in synovial sarcoma cells.

A, B. Immunoblot analyses of TFRC (A) and GPX4 (B) expression in Yamato cells expressing a scrambled or SHARPIN-specific shRNA.
