# Supplementary material for: SHARPIN Enhances Ferroptosis in Synovial Sarcoma Cells via NF-κB- and PRMT5-Mediated PGC1α Reduction
Source: Cancers (Basel). 2023 Jul 4;15(13):3484. doi: 10.3390/cancers15133484 (PMC10341212; doi:10.3390/cancers15133484)
Supplement: Supplementary file 1 [file cancers-15-03484-s001.zip › Supple FIgure11.pdf]

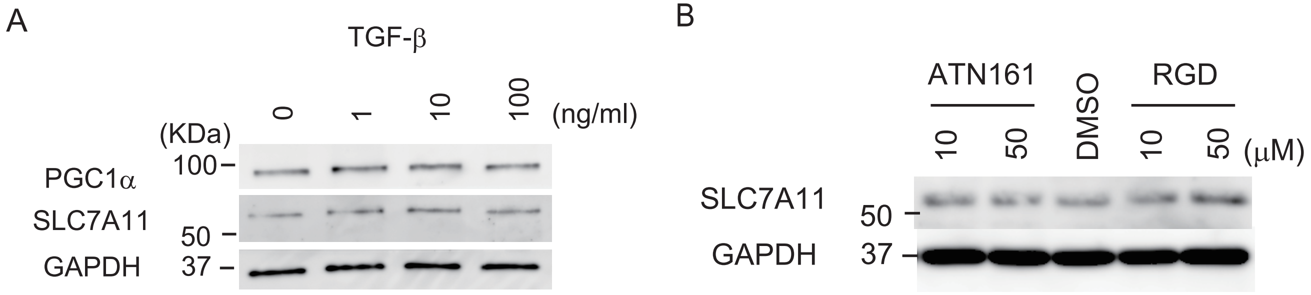

Figure S11. The expression levels of PGC1α and SLC7A11 are not altered by TGF-β stimulation, integrin inhibition (ATN-161), or integrin stimulation (RGD peptide) in Yamato cells. Cells were treated with the indicated concentrations of the inhibitors for 24 hours.
