# Supplementary material for: SHARPIN Enhances Ferroptosis in Synovial Sarcoma Cells via NF-κB- and PRMT5-Mediated PGC1α Reduction
Source: Cancers (Basel). 2023 Jul 4;15(13):3484. doi: 10.3390/cancers15133484 (PMC10341212; doi:10.3390/cancers15133484)
Supplement: Supplementary file 1 [file cancers-15-03484-s001.zip › Supple Figure3.pdf]

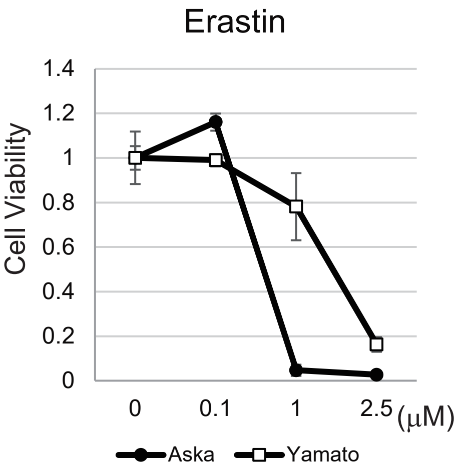

Figure S3. Viability assays of the Aska and Yamato synovial sarcoma cell lines following treatment with erastin.

The Aska (black) and Yamato (white) cell lines were treated with the indicated concentration of erastin for 24 hours.
