# Supplementary material for: SHARPIN Enhances Ferroptosis in Synovial Sarcoma Cells via NF-κB- and PRMT5-Mediated PGC1α Reduction
Source: Cancers (Basel). 2023 Jul 4;15(13):3484. doi: 10.3390/cancers15133484 (PMC10341212; doi:10.3390/cancers15133484)
Supplement: Supplementary file 1 [file cancers-15-03484-s001.zip › Supple Figure4.pdf]

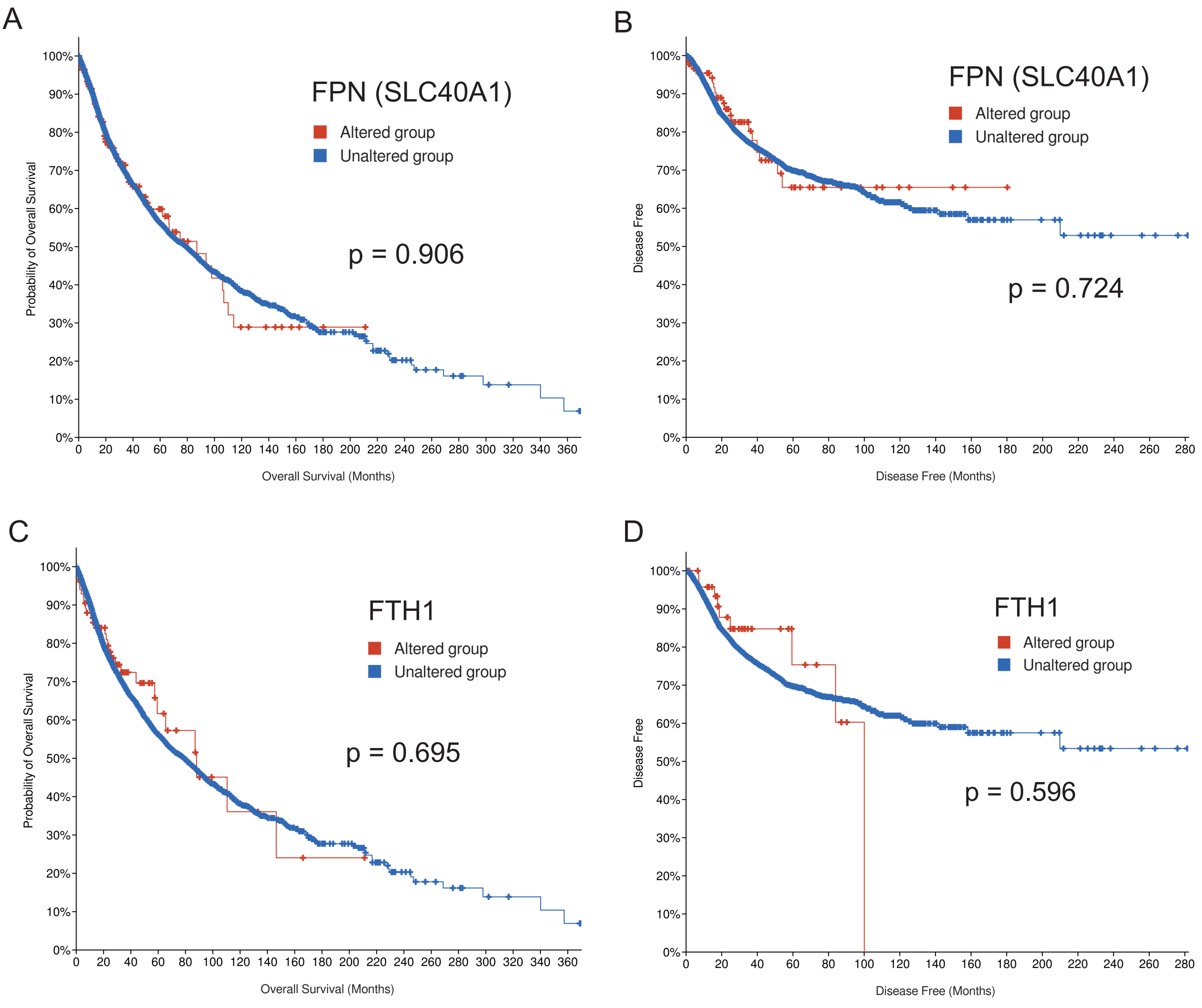

Figure S4. The expression levels of FPN and FTH1 are not related to the prognosis of cancer.

A, B. Kaplan-Meier curves showing the relationship between FPN gene amplification (AMP) and OS (A) or DFS (B), based on a TCGA Pan-Cancer Atlas Studies dataset including all types of cancer (A: n = 10,800; B: n = 5373). Subjects were dichotomized into FPN AMP and no AMP groups.

C, D. Kaplan-Meier curves showing the relationship between FTH1 gene amplification (AMP) and OS (C) or DFS (D), based on a TCGA Pan-Cancer Atlas Studies dataset including all types of cancer (C: n = 10,800; D: n = 5373). Subjects were dichotomized into FTH1 AMP and no AMP groups.

Statistical significance was calculated using a log-rank test. The P-value is indicated in each figure.
