# Supplementary material for: SHARPIN Enhances Ferroptosis in Synovial Sarcoma Cells via NF-κB- and PRMT5-Mediated PGC1α Reduction
Source: Cancers (Basel). 2023 Jul 4;15(13):3484. doi: 10.3390/cancers15133484 (PMC10341212; doi:10.3390/cancers15133484)
Supplement: Supplementary file 1 [file cancers-15-03484-s001.zip › Supple Figure6.pdf]

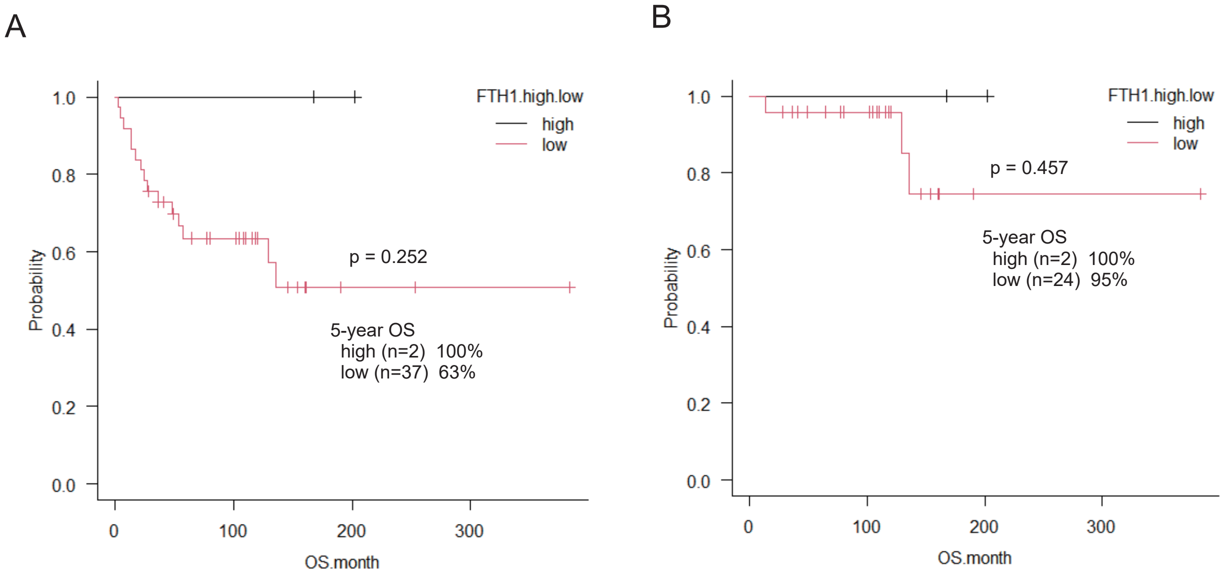

Figure S6. FTH1 expression is not significantly associated with poor prognosis of synovial sarcoma.

A, B. Kaplan-Meier curves showing the relationship between FTH1 mRNA expression levels and OS of all patients with synovial sarcoma treated in our institute (A; n = 39) or of patients with localized synovial sarcoma treated in our institute (B; n = 26). Subjects were dichotomized into FTH1 high (z-score ≥2) and FTH1 low (z-score <2) groups.

Statistical significance was calculated using a log-rank test. The P-value is indicated in each figure.
