# Supplementary material for: SHARPIN Enhances Ferroptosis in Synovial Sarcoma Cells via NF-κB- and PRMT5-Mediated PGC1α Reduction
Source: Cancers (Basel). 2023 Jul 4;15(13):3484. doi: 10.3390/cancers15133484 (PMC10341212; doi:10.3390/cancers15133484)
Supplement: Supplementary file 1 [file cancers-15-03484-s001.zip › Supple Figure8.pdf]

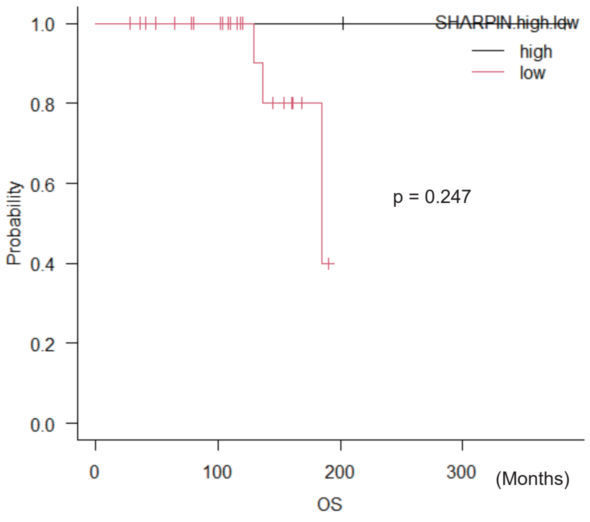

Figure S8. SHARPIN mRNA expression is not significantly associated with OS of patients with synovial sarcoma treated in our institute.

Kaplan-Meier curve showing the relationship between SHARPIN mRNA expression and OS of patients with localized synovial sarcoma treated in our institute (n = 26). Subjects were dichotomized into SHARPIN high (z-score ≥2) and SHARPIN low (z-score <2) groups. Statistical significance was calculated using a log-rank test. The P-value is indicated.
