# Supplementary material for: SHARPIN Enhances Ferroptosis in Synovial Sarcoma Cells via NF-κB- and PRMT5-Mediated PGC1α Reduction
Source: Cancers (Basel). 2023 Jul 4;15(13):3484. doi: 10.3390/cancers15133484 (PMC10341212; doi:10.3390/cancers15133484)
Supplement: Supplementary file 1 [file cancers-15-03484-s001.zip › Supple Figure9.pdf]

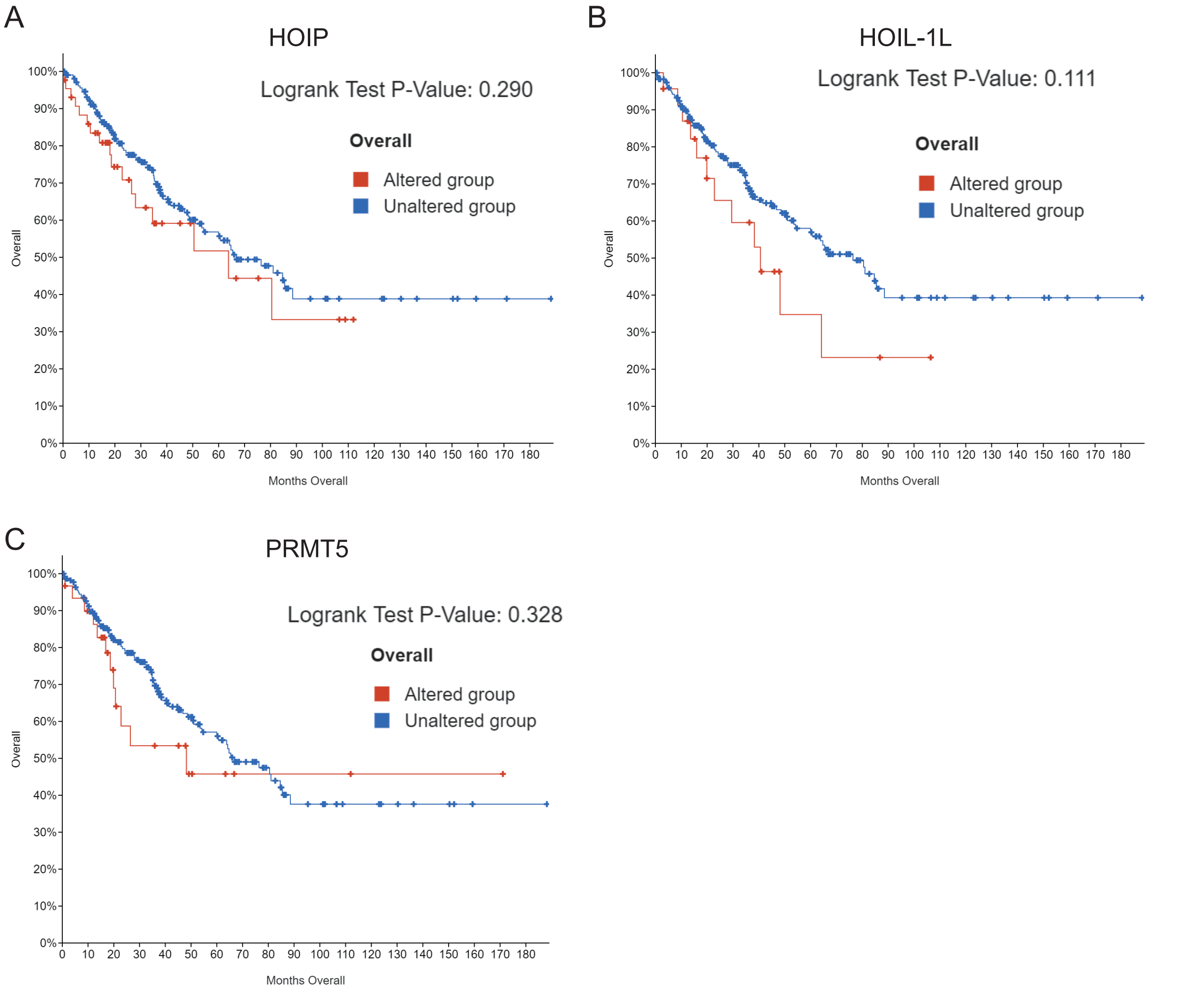

Figure S9. HOIP, HOIL-1L, and PRMT5 gene amplifications are not related to soft tissue sarcoma prognosis.

A. Kaplan-Meier curve showing the relationship between OS and HOIP gene amplification (AMP) or high mRNA expression, based on a TCGA Firehose Legacy dataset of soft tissue sarcoma samples (n = 251). Subjects were dichotomized into HOIP AMP or HIGH mRNA expression (z-score ≥2) and no AMP or LOW mRNA expression (z-score <2) groups.

B. Kaplan-Meier curve showing the relationship between OS and HOIL-1L gene amplification (AMP) or high mRNA expression, based on a TCGA Firehose Legacy dataset of soft tissue sarcoma samples (n = 251). Subjects were dichotomized into HOIL-1L AMP or HIGH mRNA expression (z-score ≥2) and no AMP or LOW mRNA expression (z-score <2) groups.

C. Kaplan-Meier curve showing the relationship between OS and PRMT5 gene amplification (AMP) and/or high mRNA expression, based on a TCGA Firehose Legacy dataset of soft tissue sarcoma samples (n = 251). Subjects were dichotomized into PRMT5 AMP or HIGH mRNA expression (z-score ≥2) and no AMP or LOW mRNA expression (z-score <2) groups.

Statistical significance was calculated using a log-rank test. The P-value is indicated in each figure.
